# Synthesis and Magnetic Properties of Stable Radical Derivatives Carrying a Phenylacetylene Unit

**DOI:** 10.3390/molecules23020371

**Published:** 2018-02-09

**Authors:** Shogo Miyashiro, Tomoaki Ishii, Youhei Miura, Naoki Yoshioka

**Affiliations:** Department of Applied Chemistry, Faculty of Science and Technology, Keio University, Yokohama 223-8522, Japan; jiro38460506@gmail.com (S.M.); tomoaki3272@gmail.com (T.I.); y-miura@applc.keio.ac.jp (Y.M.)

**Keywords:** stable radical, phenylacetylene, nitronyl nitroxide, verdazyl, magnetic property, computational analysis

## Abstract

A nitronyl nitroxide derivative, 2-phenylethynyl-4,4,5,5-tetramethyl-4,5-dihydro-1*H*-imidazol-1-oxyl-3-oxide (**1**), and two verdazyl derivatives carrying a phenylacetylene unit, 1,5-diphenyl-3-phenylethynyl-6-oxo-1,2,4,5-tetrazin-2-yl (**2**) and 1,5-diisopropyl-3-phenylethynyl-6-oxo-1,2,4,5-tetrazin-2-yl (**3**), were synthesized and their packing structures were studied by X-ray crystallographic analysis and magnetically characterized in the solid state. While **1** and **3** had an isolated doublet spin state, **2** formed an antiferromagnetically coupled pair (2*J*/*k*_B_ = −118 K). Density functional theory (DFT) calculations reveal that the spin density polarized in the phenyl group decreases as the dihedral angle between the phenyl ring and radical plane increases.

## 1. Introduction

**Chart 1:**
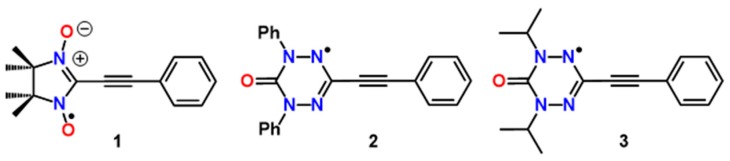
The chemical structures of **1**, **2** and **3**.

The study of stable organic radicals has attracted a great deal of attention because of their wide applicability for spin probes, molecule-based magnets, and molecular conductors [1,2,3,4,5,6,7,8]. To assess the magnetic properties of organic radical solids, it is important to investigate the correlation between molecular arrangement and magnetic properties based on crystal engineering strategies [9,10,11,12,13,14,15,16,17,18,19,20,21,22,23,24,25,26,27]. For the purpose of inducing the formation of magnetic molecular self-assemblies, van der Waals interactions [9,10,12,13,14,18], hydrogen bonds [16,17,19,20,21,22,23,25,26], and aromatic stackings [11,15,24,27] have been introduced to various stable radical derivatives such as phenoxyl, nitroxyl, etc. and their effects have been examined. As for verdazyl radical derivatives, strong intermolecular magnetic interactions induced by aromatic stackings have been reported [28,29,30,31,32,33,34,35]. In addition to the control of intermolecular interactions, introduction of specific substituents is also a useful tool for design of molecular packing. The ethynyl group has a rigid linker, and it can also propagate magnetic interaction through the conjugate system and freely rotate around the bond. In the field of crystal engineering, the free rotation of ethynyl group is widely used for the construction of a supramolecular architecture [36,37]. Although the synthetic study [38,39,40,41,42] and computational study [43,44,45] of ethynyl-substituted derivatives were reported, a limited number of papers have discussed their magneto-structural correlation [33,34,42]. We synthesized three derivatives, 2-phenylethynyl-4,4,5,5-tetramethyl-4,5-dihydro-1*H*-imidazol-1-oxyl-3-oxide (**1**), 1,5-diphenyl-3-phenylethynyl-6-oxo-1,2,4,5-tetrazin-2-yl (**2**), and 1,5-diisopropyl-3-phenylethynyl-6-oxo-1,2,4,5-tetrazin-2-yl (**3**) (Chart 1). While the synthesis of **1** has already been reported [38], structural analysis and solid-state magnetic characterization were not conducted. In this paper, we described the crystal structures and magnetic properties of **1**, **2** and **3**. To elucidate the effect of a directly substituted ethynyl group, the spin densities of **1**, **2** and **3** were estimated using a computational method.

## 2. Results

### 2.1. Synthesis and Electron Paramagnetic Resonance Spectra

**Scheme 1:**
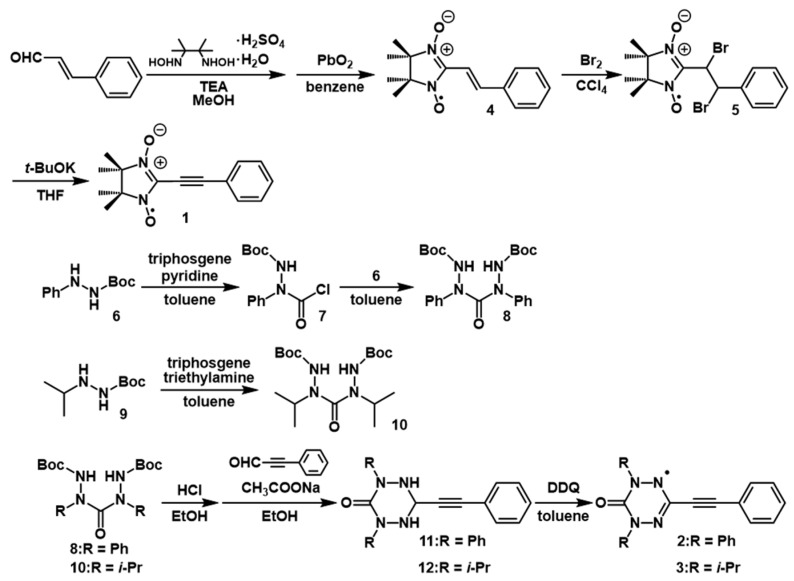
Synthesis of **1**, **2**, and **3**.

The syntheses of compounds **1**–**3** are summarized in Scheme 1. Typically, 2,3-bis(hydroxyamino)-2,3-dimethylbutane sulfate reacts with an aldehyde and the precursor of nitronyl nitroxide (NN) is obtained, but this method did not lead to **1** due to the reaction between ethynyl and the hydroxy group [40]. Therefore, the synthesis route of **1** was basically followed by Ullman’s route through alkene and dehydrobromide compounds [38]. To obtain the verdazyl (VZ) derivatives **2** and **3**, the hydrazine derivatives protected by Boc, **8** and **10** [46,47,48] were synthesized from **6** [49] and **9** [50], respectively. They were combined with the aldehyde derivative [51,52] and **2** and **3** were synthesized by the oxidation of **11** and **12**, respectively.

The solution electron paramagnetic resonance (EPR) spectra of these radicals exhibited typical patterns for NN and VZ derivatives (Figure S5). The hyperfine coupling constants (hfcc) and *g* value of compounds **1**–**3** are summarized in Table 1.

### 2.2. X-ray Structural Analysis

**Figure 1:**
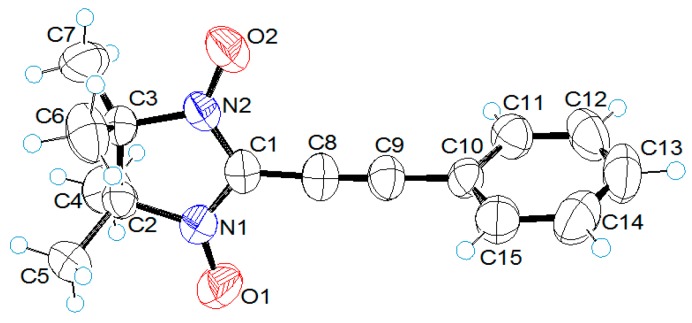
ORTEP drawing of **1**. Thermal ellipsoids are shown as 50% probability.

**Figure 2:**
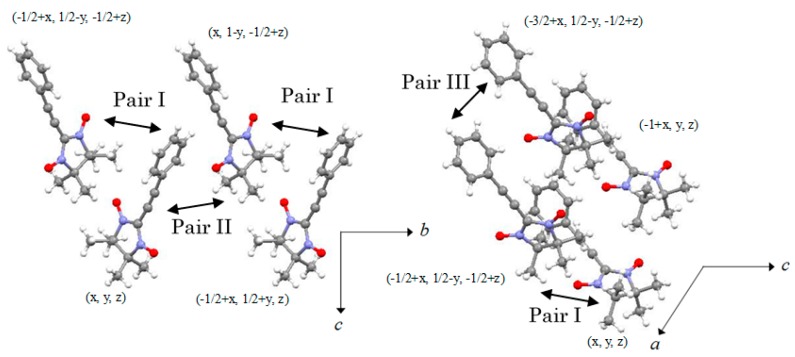
Crystal structure of **1**.

The single crystal of **1** was obtained by slow evaporation of the solvent (dichloromethane:*n*-hexane = 5:3) and it was a blue plate-like crystal. The X-ray structure analysis of **1** revealed that the crystal system was monoclinic and the space group was *Cc* (Table S1) [53]. Figure 1 shows an ORTEP drawing of **1** and the dihedral angle of the phenyl ring to the NN group was determined to be 77.81(12)°, which is a very big torsion. Three types of intermolecular close arrangements were confirmed, labeled Pair I, Pair II, and Pair III (Figure 2). In Pair I, the O atom of NN and two H atoms of the methyl group were the closest sites. On the other hand, the O atom of NN and H atom of the phenyl group were the closest to Pair II and Pair III, respectively.

**Figure 3:**
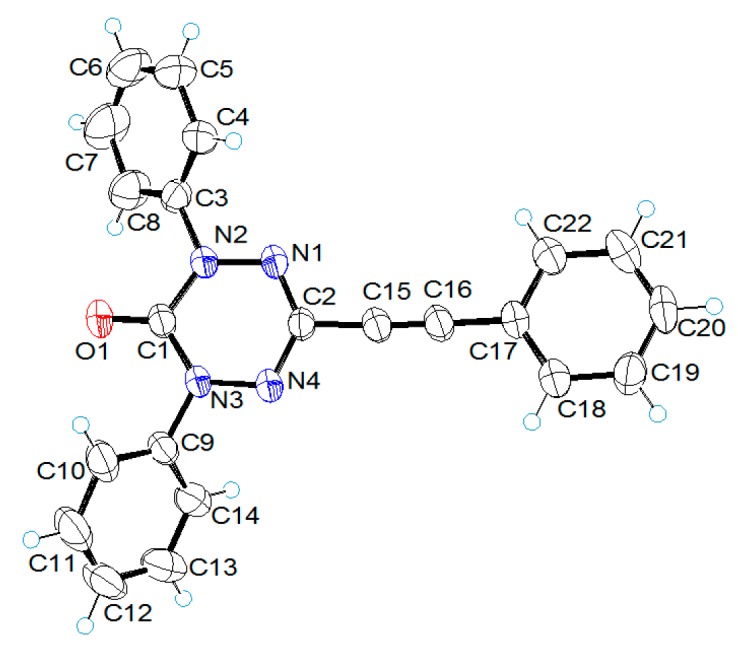
ORTEP drawing of **2**. Thermal ellipsoids are shown as 50% probability.

**Figure 4:**
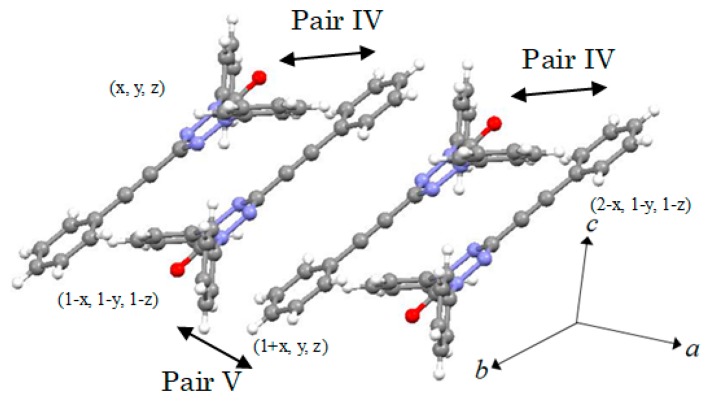
Crystal structure of **2**.

The slow evaporation of a mixed solvent (ethyl acetate:*n*-hexane = 1:1) formed the single crystal of **2** as a dark red plate-like crystal. The crystal system and space group were triclinic and *P*-1, respectively (Table S1) [53]. Figure 3 shows the ORTEP drawing of **2**. The dihedral angle between the VZ ring and phenyl group at positions 1 and 5 were 41.68(9)° and 50.08(6)°, respectively, but for the phenyl group at position 3, it was 8.26(11)°. **2** had an antiparallel stacking and two intermolecular contacts (Figure 4). The intermolecular distances of Pair IV and Pair V were 3.343(2) Å for *d*_N1-N4’_ and 3.710(3) Å for *d*_C15-C16’_.

**Figure 5:**
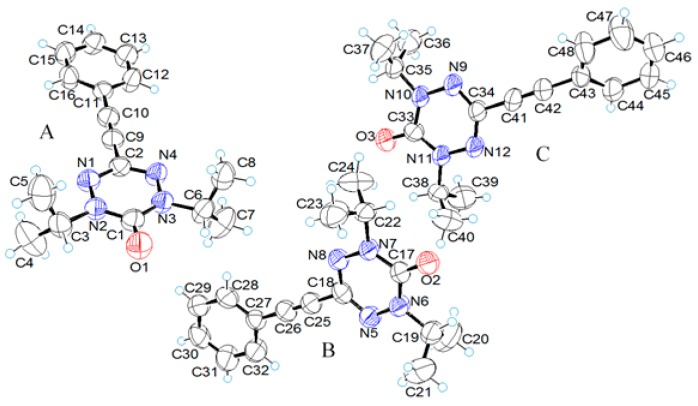
ORTEP drawing of **3**. Thermal ellipsoids are shown as 50% probability.

**Figure 6:**
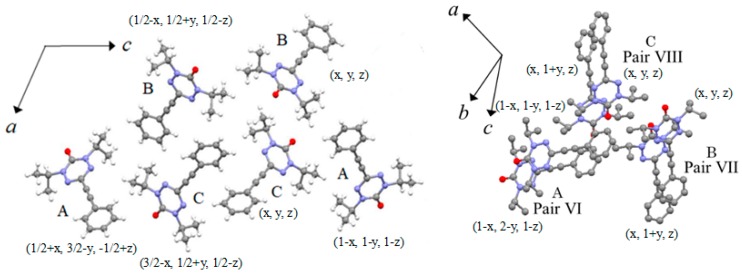
Crystal structure of **3**.

The single crystal of **3** was obtained by slow evaporation of a mixed solvent (dichloromethane:*n*-hexane = 1:2) as orange needles. The crystal system and space group were monoclinic and *P*2_1_/*n*, respectively [53]. Details of the crystallographic data are listed in Table S1. The crystal packing of **3** was composed of three different molecules labeled A, B, and C in Figure 5. Dihedral angles between the phenyl group and VZ unit were 7.42(25)° (A), 3.88(25)° (B), and 46.02(13)° (C). There was linear stacking between the same structural molecules; A, B, and C made Pair VI, Pair VII, and Pair VIII (Figure 6), respectively. The intermolecular distances were 3.701(5) Å for *d*_C2-C11’_, 3.681(6) Å for *d*_C18-C27’_, and 4.171(6) Å for *d*_C34-C43’_ in Pair VI, Pair VII, and Pair VIII, respectively.

### 2.3. Magnetic Properties

**Figure 7:**
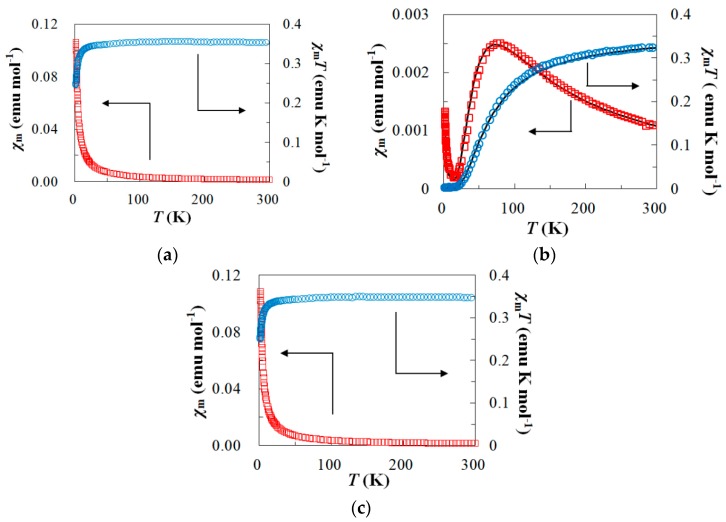
Temperature dependence of *χ*_m_*T* (○) and *χ*_m_ (□) for **1**(**a**), **2**(**b**) and **3**(**c**) under the applied field of 10000 Oe (**1** and **2**) or 5000 Oe (**3**) (1.8–300 K).

The solid-state magnetic susceptibility measurements of **1**, **2**, and **3** were carried out by a SQUID magnetometer in the temperature region of 1.8–300 K. The *χ*_m_*T*-*T* and *χ*_m_**-***T* plots for **1** and **3** (Figure 7) exhibit a similar pattern. Cooling did not cause any remarkable changes in the *χ*_m_*T* values, which indicated that they were in an isolated doublet-state. The *χ*_m_^−1^**-***T* plots of **1** and **3** showed that their Weiss temperatures were −0.13 K and −1.0 K, respectively. However, the *χ*_m_ value of **2** had a maximum at 80 K and the minimum at 15 K, and the *χ*_m_*T* value decreased with cooling, meaning that the antiferromagnetic interaction was dominant. Fitting with the antiferromagnetic dimer model revealed that the magnetic interaction, 2*J*/*k*_B_, of **2** was −118 K, containing 0.5% of the isolated component.

Antiferromagnetic dimer model equation

$$\chi_{m}=\frac{N_{A}g^{2}\mu_{B}{}^{2}}{k_{B}T}\left(\frac{1}{3+\exp\left(-\frac{2J}{k_{B}T}\right)}\left(1-\rho \right)+\frac{1}{4}\rho \right)$$

*χ*_m_: magnetic susceptibility; *N*_A_: Avogadro constant; *g*: *g* factor; *μ*_B_: Bohr magneton; 

*k*_B_: Boltzmann constant; *ρ*: isolated component.

## 3. Discussion

The magnetic interactions of the abovementioned pairs were calculated by DFT at the UB3LYP/6-31G* level, and the calculation results are substituted into Yamaguchi’s equation [54] (Table 2). In **1** and **3**, the value of *J*/*k*_B_ for all the pairs was nearly zero; these results agreed with the SQUID measurement. In these pairs, the C, N, and O atoms of the radical ring that have a large spin density were not close to the C, N, and O atoms of the neighboring radical ring. However, Pair V had a low value of *J*/*k*_B_, and Pair IV had a large negative value that was thought to cause the antiferromagnetic behavior of the SQUID measurement. It was suggested that the antiferromagnetic interaction was caused by the intermolecular interaction between the N1 and N4 atoms (*x*, *y*, *z*) that have large spin density and the N1’ and N4’ atoms of neighboring molecules (1−*x*, 1−*y*, 1−*z*). The values of the spin density of the atoms that form the six-membered ring and phenylacetylene group of **1**–**3** are shown in Tables S2–S6, respectively.

**Figure 8:**
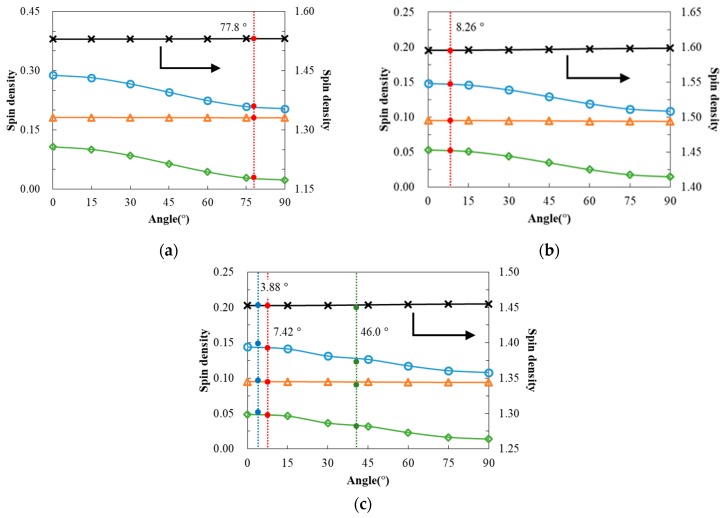
Correlation between dihedral angle and spin density of **1**(**a**), **2**(**b**) and **3**(**c**); Radical group(×), the sum of the ethynyl and phenyl(○), ethynyl(△), phenyl(◇) and experimental data(●, ● and ●).

To estimate the influence of the rotation of the ethynyl group on the extent of the spin density, the molecules that were based on the crystal structures and rotated between the phenyl group and radical ring at 15-degree intervals (0–90°) were calculated by DFT. The sum of the spin density that was taken as an absolute value was used in the plots of Figure 8. The spin density of the radical group and the ethynyl group remained almost constant; however, that of the phenyl group decreased with rotation. This result indicated that the spin density was difficult to expand at the higher dihedral angle of ethynyl like other conjugated systems.

## 4. Experimental

Compounds **1**, **4**, and **5** [38], **6** [49], **9** and **10** [50], phenylpropargylaldehyde [48] were synthesized with reference to the respective papers. The details of the methods for synthesizing the other compounds are summarized in the Supplementary Materials.

The NMR spectra were measured by JEOL JNM-LA300 (Tokyo, Japan) (tetramethylsilane was used as the reference). Mass spectroscopy was carried out using a Bruker Ultraflex II (MALDI-TOF, with sinapic acid used as the matrix) (Billerica, MA, USA). The IR spectra were measured using a JASCO FT/IR-4100 (Tokyo, Japan). The crystal data were collected using a Bruker D8 Venture with Mo–Kα radiation (0.71073 Å). The structures were solved by the direct method using SHELXT-2013 (Göttingen, Germany) [55] and refined by *F*^2^ full matrix least squares using SHELXL-2014 (Göttingen, Germany) [56] in the Bruker APEX-II program package. The magnetic susceptibility measurement was carried out using a Quantum Design MPMS-XL SQUID (San Diego, CA, USA) magnetometer in the temperature range of 1.8–300 K under the applied field of 10000 Oe (**1** and **2**) or 5000 Oe (**3**). The EPR spectra were recorded using a Bruker E500 spectrometer (Billerica, MA, USA) at room temperature. The EPR data were simulated by the Winsim ver. 0.96 program (Bethesda, MD, USA) [57]. The DFT calculation was carried out using the Gaussian 09 program (Wallingford, CT, USA) [58].

## 5. Conclusions

As stable organic radicals carrying an ethynyl group, the nitronyl nitroxide derivative **1** and verdazyl derivatives **2** and **3** were synthesized. X-ray crystal analysis revealed their packing structures, and **1**–**3** had several types of close intermolecular contacts. The magnetic susceptibility measurement revealed that **1** and **3** had an isolated doublet-state and **2** was an antiferromagnetic compound that fitted with 2*J*/*k*_B_ = −118 K containing 0.5% of the isolated component. DFT calculations of **1**, **2**, and **3** agreed with the results of the magnetic susceptibility measurements, and indicated that the spin density of the phenyl group decreased due to the free rotation of the acetylene unit.

## Figures and Tables

**Table 1:** EPR parameters of **1**, **2** and **3** in toluene.

	*α*_N_(G)	*g*
**1**	7.32, 7.35	2.0062
**2**	4.62, 4.64, 6.39, 6.40	2.0033
**3**	5.29, 5.61, 6.52, 6.56	2.0031

**Table 2:** Estimation of intermolecular magnetic interactions of **1**, **2**, and **3**.

	1			2		3		
	Pair I	Pair II	Pair III	Pair IV	Pair V	Pair VI	Pair VII	Pair VIII
2*J*/*k*_B_ (K)	0.190	0.190	−0.0632	−79.4	−1.58	−1.26	−2.91	−0.948

## Supplementary Materials

The Supplementary Materials are available online: supplementary materials contain synthetic procedure, EPR spectra, crystallographic parameter, and distributions of SOMO and spin density.
