# Supplementary material for: Synthesis and Magnetic Properties of Stable Radical Derivatives Carrying a Phenylacetylene Unit
Source: Molecules. 2018 Feb 9;23(2):371. doi: 10.3390/molecules23020371 (PMC6017151; doi:10.3390/molecules23020371)
Supplement: Supplementary file 1 [file molecules-23-00371-s001.pdf]

Supporting Information

Synthesis and Magnetic Properties of Stable Radical Derivatives Carrying a Phenylacetylene Unit

Shogo Miyashiro, Tomoaki Ishii, Youhei Miura, and

Naoki Yoshioka*

Department of Applied Chemistry, Faculty of Science and Technology, Keio University, Yokohama 223-8522, Japan

E-mail: yoshioka@applc.keio.ac.jp

Table of contents

Synthetic procedure S2

EPR spectra S7

Crystallographic parameter S8

Distributions of SOMO and spin density S9

Synthetic procedure

tert-butyl 2-phenyl-2-chlorocarbonylhydrazinecarboxylate **7**

To a solution of **6** (1.04 g, 5.0 mmol) in toluene, pyridine (400 µL, 5.0 mmol, 1.0 eq.) was added at –78 °C under N_2. A solution of triphosgene (740 mg, 2.5 mmol, 0.50 eq.) in toluene was added slowly. The mixture was stirred at room temperature for 16 h, and the solvent was removed under vacuum. The solid was diluted with water and AcOEt, and the organic layer was dried over Na_2SO_4 and evaporated under vacuum. The white residue was purified by column chromatography on silica gel using AcOEt : *n*-hexane = 1 : 4 as the eluent to afford a white solid. Yield: 1.16 g, 86%; R_f value: 0.3 (AcOEt : *n*-hexane = 1 : 4); mp: 96-98 °C; ^{1}H-NMR ($CDCl_3$, 300 MHz) δ (ppm): 7.45-7.41 (m, 5H), 1.50 (s, 9H); ^{13}C-NMR ($CDCl_3$, 75 MHz) δ (ppm): 154.0, 140.9, 129.1, 127.7, 124.1, 123.7, 82.7, 28.0; IR (KBr pellet, cm^{-1}): 3275 ($\nu_{N\text{-}H}$), 1759 ($\nu_{C=O}$), 1709 ($\nu_{C=O}$).

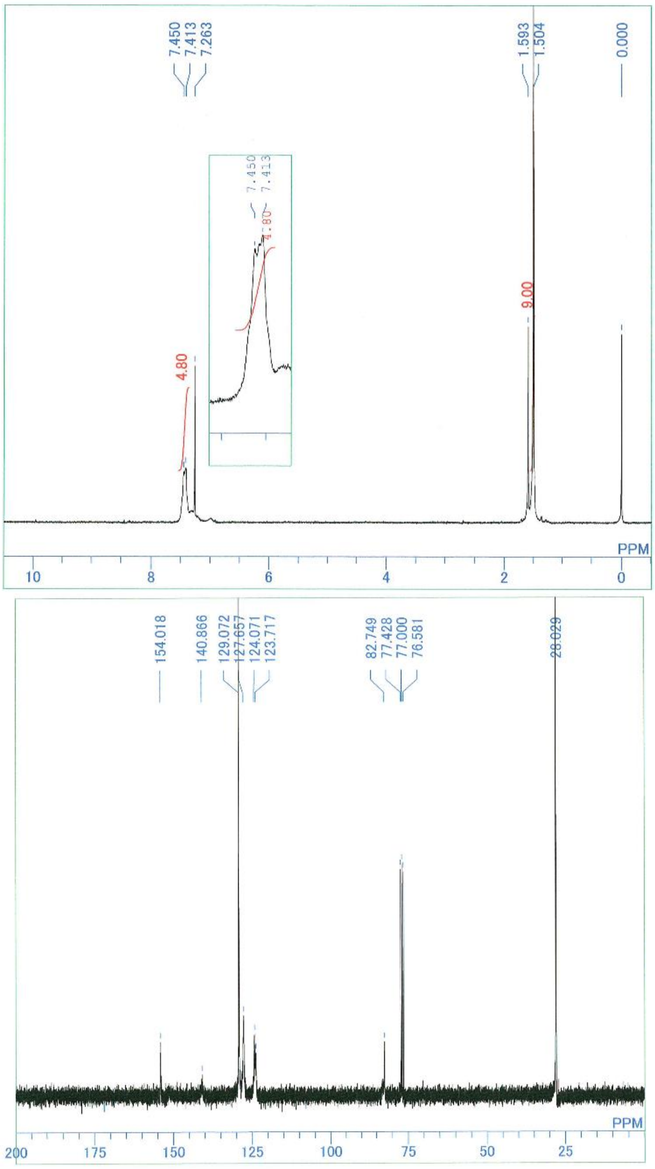

Figure S1. ^{1}H and ^{13}C NMR spectra of **7**.

di-*tert*-butyl-2, 2'-cabonylbis-(2-phenylhydrazinecarboxylate) **8**

A mixture of **7** (1.16 g, 4.3 mmol) and **6** (1.79 g, 8.6 mmol, 2.0 eq.) was solved in toluene under N_2. The solution was stirred at 110 °C for 16 h, and water and AcOEt were added to the solution. The organic layer was dried over Na_2SO_4 and evaporated under vacuum. The residue was purified by column chromatography on silica gel using AcOEt : *n*-hexane = 1 : 2 to afford a white solid. Yield: 1.33 g, 70%; R_f value: 0.5 (AcOEt : *n*-hexane = 1 : 2); mp: 178-179 °C; ^{1}H-NMR (aceton-d_6, 300 MHz) δ (ppm): 7.34 (d, J = 6.6 Hz, 4H), 7.08 (t, J = 6.9 Hz, 4H), 6.92 (t, J = 6.6 Hz, 2H), 1.49 (s, 18H); ^{13}C-NMR ($CDCl_3$, 75 MHz) δ (ppm): 157.5, 155.8, 142.6, 128.4, 125.8, 124.4, 81.8, 28.2; LRMS (MALDI-TOF): m/z = 441 [M-H]; IR (KBr pellet, cm^{-1}): 3327 (ν_{N-H}), 1714 ($\nu_{C=O}$).

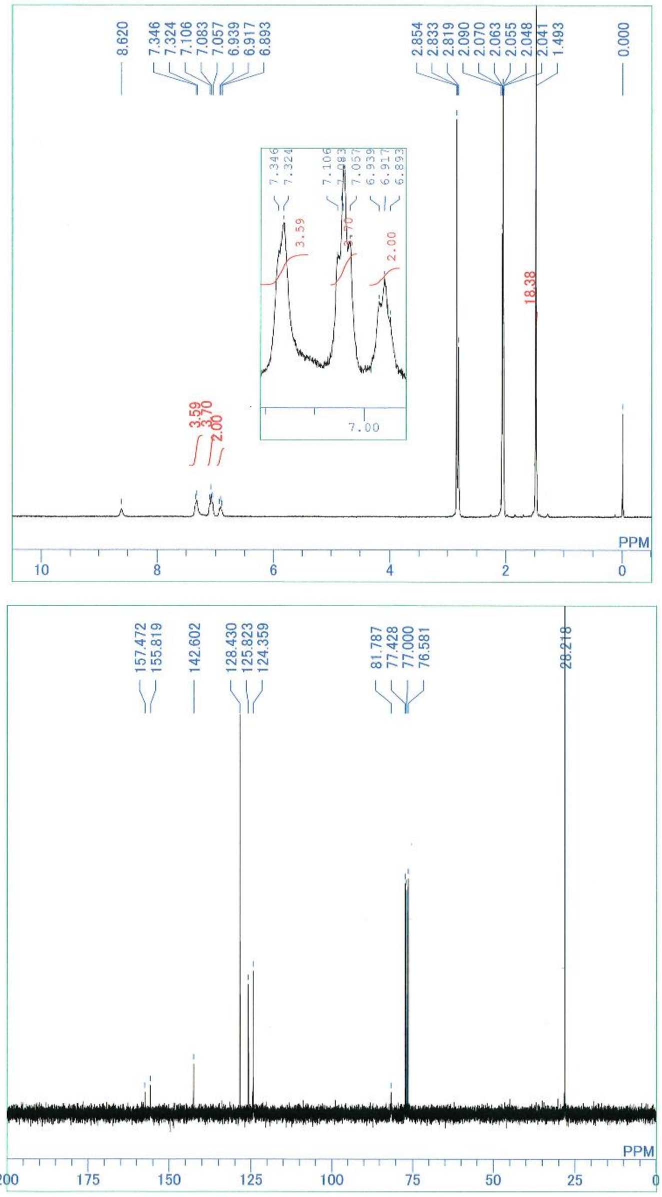

Figure S2. ^{1}H and ^{13}C NMR spectra of **8**.

2,4-diphenyl-6-(phenylethynyl)-1,2,4,5-tetrazinan-3-one **11**

To a solution of **8** (1.33 g, 3.0 mmol) in 33 mL of EtOH, 17 mL of 1M HCl was added. The mixture was stirred at 80 °C for 1 h, and then the mixture was stirred at room temperature for 16 h. The solvent was removed under vacuum, and the solid was washed with $CHCl_3$, then the white residue (863 mg, 2.7 mmol) was obtained. To a solution of the white residue (123 mg, 0.39 mmol) in EtOH, 3-phenylpropiolaldehyde (51 mg, 0.39 mmol, 1.0 eq.) and sodium acetate (64 mg, 0.78 mmol, 2.0 eq.) were added under N_2. The mixture was stirred for 24 h, and the solvent was removed under vacuum. Water and CH_2Cl_2 were added to the residue and the organic layer was dried over Na_2SO_4 and evaporated under vacuum. The yellow residue was purified by column chromatography on silica gel using CH_2Cl_2 as the eluent to afford a white solid. Yield: 106 mg, 76%; R_f value: 0.1 (CH_2Cl_2); mp: 195-198 °C; ^{1}H-NMR ($CDCl_3$, 300 MHz) δ (ppm): 7.68-7.61 (m, 5H), 7.45-7.30 (m, 8H), 7.17-7.12 (m, 2H), 5.35 (t, J = 8.7 Hz, 1H), 4.93 (d, J = 8.7 Hz, 2H); ^{13}C-NMR (DMSO, 75 MHz) δ (ppm): 155.0, 143.0, 131.4, 129.1, 128.8, 128.0, 123.8, 122.2, 121.3, 85.4, 83.9, 62.1; LRMS (MALDI-TOF): m/z = 355 [M+H]; IR (KBr pellet, cm^{-1}): 3249 ($\nu_{N\text{-}H}$), 1623 ($\nu_{C=O}$).

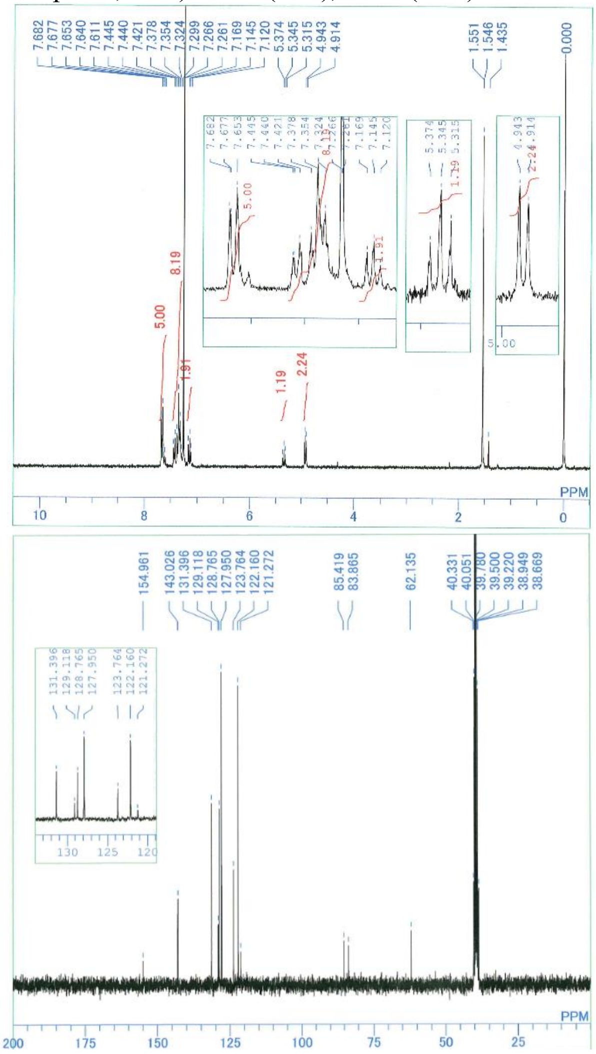

Figure S3. ^{1}H and ^{13}C NMR spectra of **11**.

1,5-diphenyl-3-phenylethynyl-6-oxo-1,2,4,5-tetrazin-2-yl **2**

To a solution of **11** (106 mg, 0.30 mmol) in toluene, DDQ (102 mg, 0.45 mmol, 1.5 eq.) was added under N_2. The mixture was stirred for 1 h, and the solvent was removed under vacuum. The solid was diluted with water and AcOEt, and the organic layer was dried over Na_2SO_4 and evaporated under vacuum. The residue was purified by column chromatography on silica gel using CH_2Cl_2 : *n*-hexane = 1 : 2 as the eluent to afford a red solid (88 mg, 83%). Yield: 88 mg, 83%; R_f value: 0.5 (CH_2Cl_2 : *n*-hexane = 1 : 2); mp: 186-188 °C; LRMS (MALDI-TOF): m/z = 351 [M]; IR (KBr pellet, cm^{-1}): 2227 ($\nu_{C\equiv C}$), 1702 ($\nu_{C=O}$); Elem. Anal.: calcd. for $C_{22}H_{15}N_4O$: C, 75.20; H, 4.30; N, 15.94. Found: C, 75.29; H, 4.29; N, 15.59.

2,4-diisopropyl-6-(phenylethynyl)-1,2,4,5-tetrazinan-3-one **12**

To a solution of **10** (3.03 g, 8.1 mmol) in 40 mL of EtOH, 20 mL of 1M HCl was added. The mixture was stirred at 80 °C for 30 min, and then the mixture was stirred at room temperature for 16 h. The solvent was removed under vacuum, and the solid was washed with $CHCl_3$, then the white residue (1.95 g, 7.9 mmol) was obtained. To a solution of the white residue (235 mg, 0.95 mmol, 1.3 eq.) in 3 mL of EtOH, 3-phenylpropiolaldehyde (95 mg, 0.73 mmol) and sodium acetate (156 mg, 1.9 mmol, 2.0 eq.) were added under N_2. The mixture was stirred for 24 h, and the solvent was removed under vacuum. Water and AcOEt were added to the residue and the organic layer was dried over Na_2SO_4 and evaporated under vacuum. The yellow residue was purified by column chromatography on silica gel using AcOEt : *n*-hexane = 1 : 1 as the eluent to afford a white solid. Yield: 160 mg, 77%; R_f value: 0.3 (AcOEt : *n*-hexane = 1 : 1); mp: 151.5-153 °C; ^{1}H-NMR ($CDCl_3$, 300 MHz) δ (ppm): 7.46 (d, J = 8.1 Hz, 2H), 7.37-7.32 (m, 3H), 4.67-4.58 (m, 3H), 3.97 (d, J = 9.9 Hz, 2H), 1.16 (d, J = 6.9 Hz, 6H), 1.13 (d, J = 6.6 Hz, 6H); ^{13}C-NMR (DMSO, 75 MHz) δ (ppm): 153.9, 131.8, 129.1, 128.4, 121.4, 85.9, 82.4, 62.2, 47.6, 19.3, 18.7; LRMS (MALDI-TOF): m/z = 288 [M+2H]; IR (KBr pellet, cm^{-1}): 2243 ($\nu_{C\equiv C}$), 1583 ($\nu_{C=O}$).

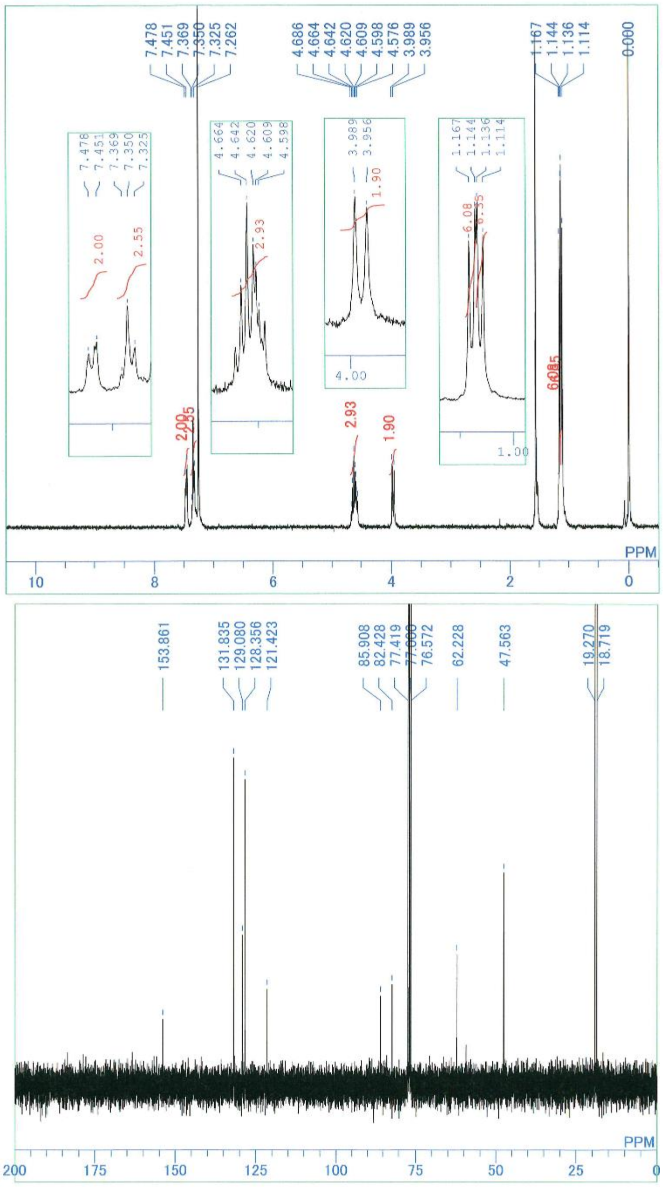

Figure S4. ^{1}H and ^{13}C NMR spectra of **12**.

1,5-diisopropyl-3-phenylethynyl-6-oxo-1,2,4,5-tetrazin-2-yl **3**

To a solution of **12** (80 mg, 0.28 mmol) in 3 mL of toluene, DDQ (95 mg, 0.42 mmol, 1.5 eq.) was added under N_2. The mixture was stirred for 2 h, and the solvent was removed under vacuum. The solid was diluted with water and AcOEt and the organic layer was dried over Na_2SO_4. The solvent was evaporated under vacuum and the residue was purified by column chromatography on silica gel using AcOEt : *n*-hexane = 1 : 3 as the eluent to afford a orange solid. Yield: 43 mg, 54%; R_f value: 0.7 (AcOEt : *n*-hexane = 1 : 3); mp: 133-135 °C; LRMS (MALDI-TOF): m/z = 285 [M+2H]; IR (KBr pellet, cm^{-1}): 2229 ($\nu_{C\equiv C}$), 1680 ($\nu_{C=O}$); Elem. Anal.: calcd. for $C_{16}H_{19}N_4O$: C, 67.82; H, 6.76; N, 19.77. Found: C, 67.03; H, 6.81; N, 19.60.

EPR spectra

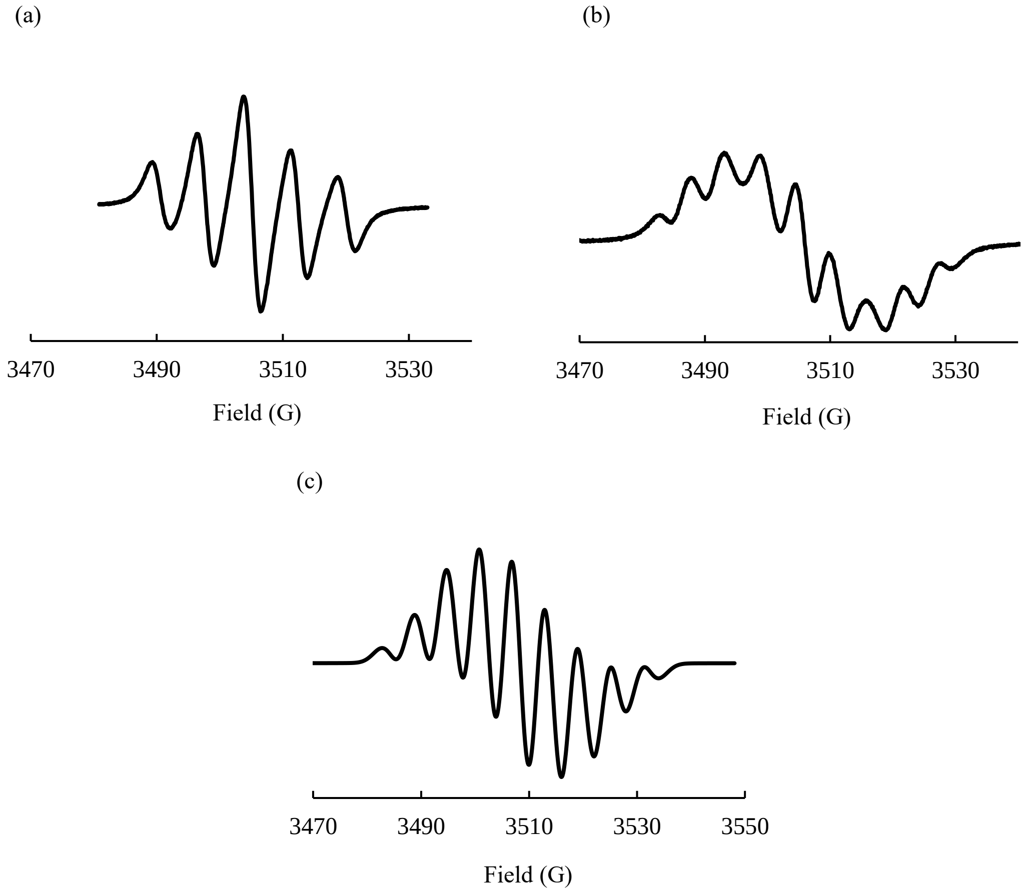

Figure S5. EPR spectra of **1**(a), **2**(b), and **3**(c) at r.t. in toluene.

Crystallographic parameter

Table S1. Crystallographic parameters of **1**, **2** and **3**.

Formula	$C_{15}H_{17}N_2O_2$(**1**)	$C_{22}H_{15}N_4O$(**2**)	$C_{16}H_{19}N_4O$(**3**)
M	257.30	351.38	283.35
Crystal system	monoclinic	triclinic	monoclinic
Space group	*Cc*	*P*-1	$P2_1/n$
a, Å	6.2033(7)	8.9743(7)	16.302(3)
b, Å	21.0864(19)	10.2833(7)	5.4709(11)
c, Å	11.1658(11)	10.9480(8)	53.971(11)
α, deg	90	102.002(2)	90
β, deg	106.122(4)	91.037(3)	97.290(5)
γ, deg	90	113.571(2)	90
V, Å^3	1403.1(2)	900.00(11)	4774.6(16)
Z	4	2	12
D, g cm^{-3}	1.218	1.297	1.183
R (I >2σ(I))	0.0423	0.0610	0.0725

Distributions of SOMO and spin density

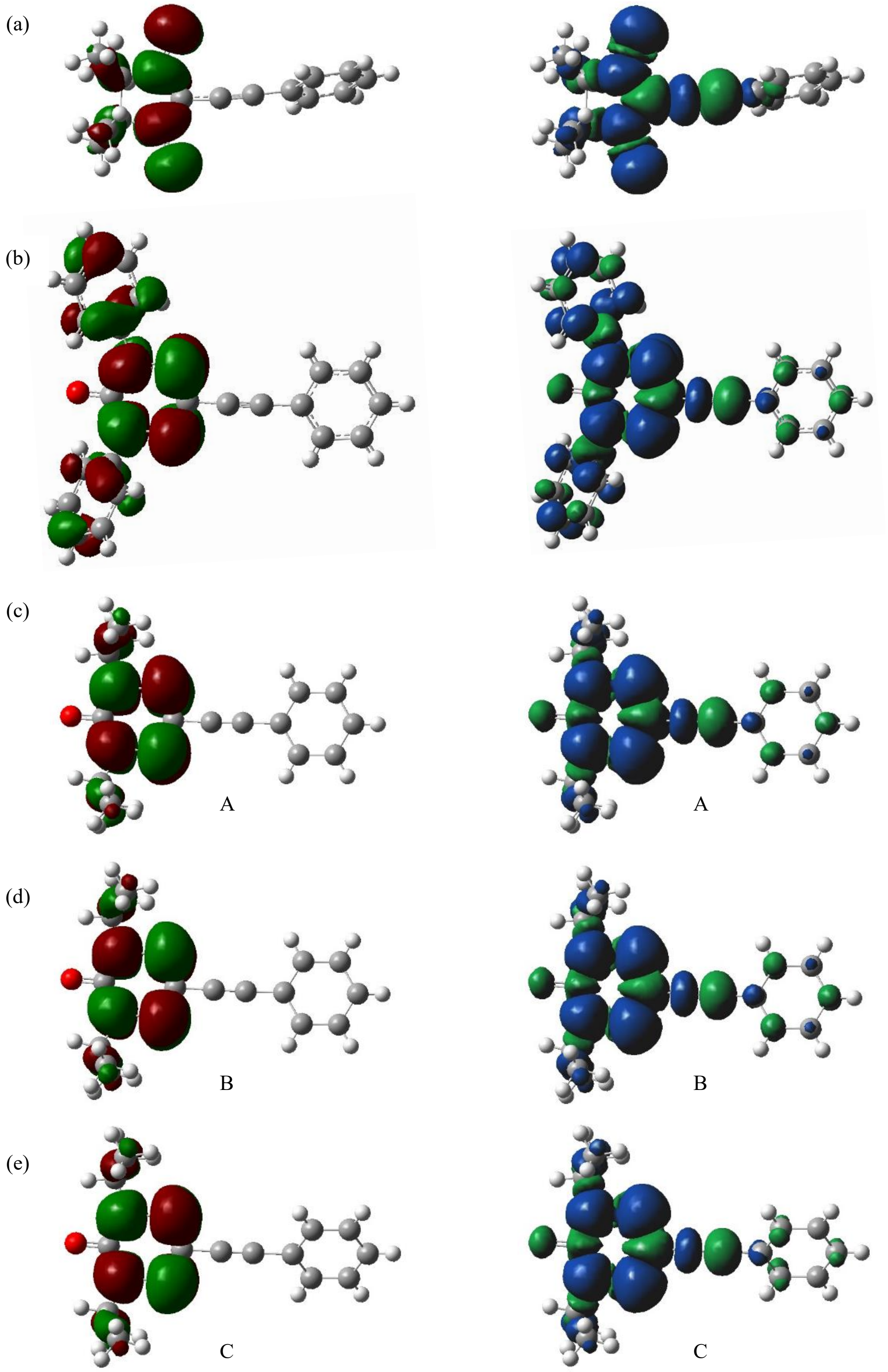

Figure S6. SOMO and spin density distributions of **1**(a), **2**(b) and **3**(c, d, e).

Table S2. The spin density distribution on the atom of **1**.

atom	spin density	atom	Spin density
N1	0.259	C9	-0.153
N2	0.264	C10	0.015
O1	0.357	C11	-0.010
O2	0.363	C12	0.003
C1	-0.220	C13	-0.006
C2	-0.015	C14	0.003
C3	-0.015	C15	-0.010
C8	0.091		

Table S3. The spin density distribution on the atom of **2**.

atom	spin density	atom	Spin density
N1	0.427	C16	-0.058
N2	0.162	C17	0.013
N3	0.158	C18	-0.012
N4	0.445	C19	0.006
O1	-0.149	C20	-0.010
C1	-0.018	C21	0.006
C2	-0.177	C22	-0.012
C15	0.045		

Table S4. The spin density distribution on the atom of **3**(A).

atom	spin density	atom	Spin density
N1	0.399	C10	-0.057
N2	0.209	C11	0.011
N3	0.210	C12	-0.010
N4	0.381	C13	0.005
O1	-0.012	C14	-0.009
C1	-0.032	C15	0.005
C2	-0.144	C16	-0.010
C9	0.038		

Table S5. The spin density distribution on the atom of **3**(B).

atom	spin density	atom	Spin density
N5	0.383	C26	-0.058
N6	0.206	C27	0.011
N7	0.214	C28	-0.010
N8	0.398	C29	0.005
O2	-0.011	C30	-0.009
C17	-0.032	C31	0.005
C18	-0.146	C32	-0.011
C25	0.039		

Table S6. The spin density distribution on the atom of **3**(C).

atom	spin density	atom	Spin density
N9	0.375	C42	-0.056
N10	0.203	C43	0.008
N11	0.218	C44	-0.006
N12	0.402	C45	0.003
O3	-0.012	C46	-0.006
C33	-0.032	C47	0.003
C34	-0.143	C48	-0.006
C41	0.035		
